# Overview on the Infections Related to Rare *Candida* Species

**DOI:** 10.3390/pathogens11090963

**Published:** 2022-08-24

**Authors:** Sunil Kumar, Awanish Kumar, Maryam Roudbary, Rasoul Mohammadi, Lucia Černáková, Célia Fortuna Rodrigues

**Affiliations:** 1 Faculty of Biosciences, Institute of Biosciences and Technology, Shri Ramswaroop Memorial University, Barabanki 225003, Uttar Pradesh, India; 2 Department of Biotechnology, National Institute of Technology, Raipur 492010, Chhattisgarh, India; 3 Department of Parasitology and Mycology, School of Medicine, Iran University of Medical Sciences, Tehran 1449614535, Iran; 4 Department of Medical Parasitology and Mycology, Infectious Diseases and Tropical Medicine Research Center, School of Medicine, Isfahan University of Medical Sciences, Isfahan 8174673461, Iran; 5 Department of Microbiology and Virology, Faculty of Natural Sciences, Comenius University in Bratislava, Ilkovičova 6, 842 15 Bratislava, Slovakia; 6 TOXRUN—Toxicology Research Unit, CESPU—Cooperativa de Ensino Superior Politécnico e Universitário, 4585-116 Gandra PRD, Portugal; 7 LEPABE—Laboratory for Process Engineering, Environment, Biotechnology and Energy, Faculty of Engineering, University of Porto, Rua Dr. Roberto Frias, 4200-465 Porto, Portugal; 8 ALiCE—Associate Laboratory in Chemical Engineering, Faculty of Engineering, University of Porto, Rua Dr. Roberto Frias, 4200-465 Porto, Portugal

**Keywords:** *Candida kefyr*, *Candida lusitaniae*, *Candida famata*, *Candida guilliermondii*, *Candida rugosa*, *Candida nivariensis*, *Candida lipolytica*, *Candida bracarensis*, *Candida africana*, *Candida blankii*, *Candida pulcherrima*

## Abstract

Atypical *Candida* spp. infections are rising, mostly due to the increasing numbers of immunocompromised patients. The most common *Candida* spp. is still *Candida albicans*; however, in the last decades, there has been an increase in non-*Candida albicans Candida* species infections (e.g., *Candida glabrata*, *Candida parapsilosis*, and *Candida tropicalis*). Furthermore, in the last 10 years, the reports on uncommon yeasts, such as *Candida lusitaniae*, *Candida intermedia,* or *Candida norvegensis*, have also worryingly increased. This review summarizes the information, mostly related to the last decade, regarding the infections, diagnosis, treatment, and resistance of these uncommon *Candida* species. In general, there has been an increase in the number of articles associated with the incidence of these species. Additionally, in several cases, there was a suggestive antifungal resistance, particularly with azoles, which is troublesome for therapeutic success.

## 1. Introduction

Deaths from fungal diseases may be five to six times higher than what is actually reported and may yield an economic burden of USD $24.3 billion [1]. Although most, but not all, yeasts belonging to the *Candida* genus are commensal microorganisms, they are commonly linked to superficial or candidemia infections [2,3], being associated with high mortality and morbidity rates [4,5]. Indeed, *Candida albicans* is still the most common *Candida* spp. described in candidiasis; however, non-*Candida albicans Candida* species (NCACs) have been rising [6,7]. *Candida albicans*, *Candida glabrata* (updated nomenclature *Nakaseomyces glabrataa* [8,9]), *Candida tropicalis*, *Candida parapsilosis,* and *Candida krusei* (updated nomenclature: *Pichia kudriavzevii* [8,9]) are responsible for, approximately, 9 out of 10 fungal infections [3,10]. Notably, in the last decade, emerging and atypical yeasts have been reported as an increased cause of fungal infections in immunocompromised and/or hospitalized patients [11,12]. Preventing or treating these infections successfully requires nationwide epidemiological and etiological data [13].

CHROMagar™ *Candida*, Polimerase Chain Reaction (PCR), or Matrix-Assisted Laser Desorption/Ionisation Time-Of-Flight Mass Spectrometry (MALDI-TOF MS) are commonly used to quickly identify yeast species [14]. With regards to the antibiotic susceptibility tests, the Minimal Inhibitory Concentrations (MIC) of antifungals (e.g., amphotericin B, 5-fluorocytosine, fluconazole, and caspofungin) must be determined according to the reference document (European Committee on Antimicrobial Susceptibility Testing, EUCAST [15], or Clinical and Laboratory Standards Institute, CLSI [16], guidelines) [14]. For most rare *Candida* spp., there is a lack of information on clinical breakpoints and antifungal susceptibility profiles. This is a serious issue in clinical practice, for both interpretations and for choosing the appropriate therapy [17,18]. Nonetheless, it has been described that the prevalence of these non-common *Candida* spp. and their susceptibility profiles can differ; therefore, their correct identification is critical [10]. The use of molecular methods (e.g., PCR) [10]. or retrospective reexamination of isolates help can reveal ambiguous species [14].

For any fungal infection, it is considered that the reference methodologies for the susceptibility tests are strongly recommended and important to guide antifungal therapy. Additionally, individualized approaches should also be considered [17,19].

This review intends to gather reported information on candidiasis related to atypical *Candida* spp. from the last decade. For this, we used the National Library of Medicine website (NIH, PubMed^®^—Medline), the words “Candida” + “infection” + “*x*”—*x* meaning “*kefyr*”, “*norvegensis*”, “*inconspicua*”, “*famata*”, “*guilliermondii*”, “*lipolytica*”, “*rugosa*”, “*pararugosa*”, “*lusitaniae*”, “*pelliculosa*”, “*nivariensis*”, “*bracarensis*”, “*intermedia*”, “*africana*”, “*blankii*”, and “*pulcherrima*” using the NCBI filters, mostly for years 2011–2021. Non-*C. albicans*, non-*C. parapsilosis* complex, non-*C. glabrata* complex, non-C. *tropicalis*, and non-*C. krusei* which have been particularly critical in terms of candidiasis the last years were included in this review.

## 2. Uncommon *Candida* spp.: Infections, Treatment, and Resistance

**Figure 1:**
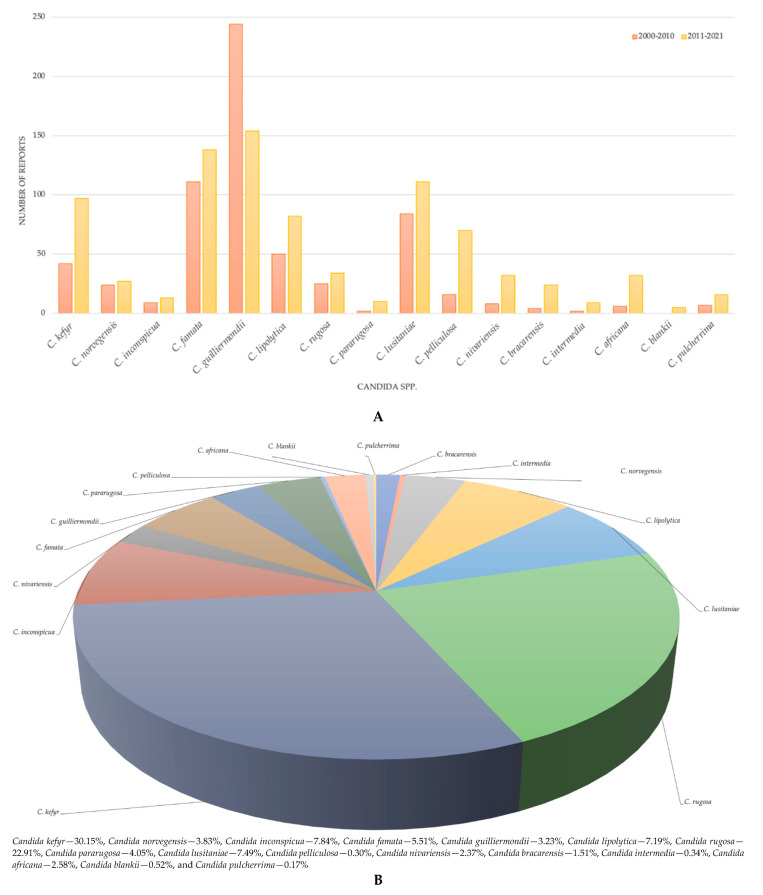
(**A**) Number of reports published between 2000 and 2010 and 2011 and 2021 associated with rare *Candida* sp. (**B**) Data/Statistics: (**A**) the number of reports determined using PubMed^®^ filters; (**B**) percentages calculated from the *n* of each rare *Candida* species and *n* of all rare *Candida* species considered in this review, the reports between 2011 and 2021, according to the search made in NIH and PubMed^®^—Medline.

**Figure 2:**
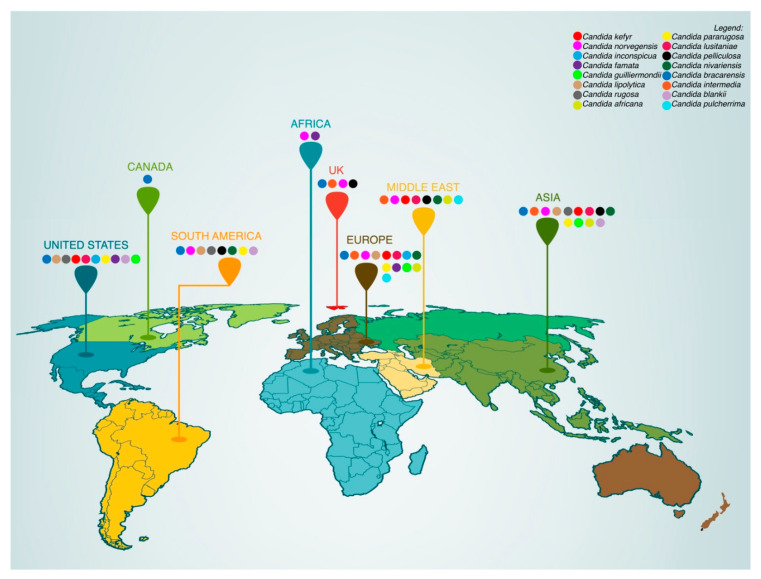
Worldwide incidence distribution of rare *Candida* species. (Data/Statistics: countries reported in papers published between 2011 and 2021, according to the search made in NIH and PubMed^®^—Medline).

There have been several reports describing the association of uncommon NCACs to fungal infections. Among them, *Candida kefyr*, *Candida norvegensis*, *Candida inconspicua*, *Candida famata*, *Candida guilliermondii*, *Candida lipolytica*, *Candida rugosa*, *Candida pararugosa*, *Candida lusitaniae*, *Candida pelliculosa*, *Candida nivariensis*, *Candida bracarensis*, *Candida intermedia*, *Candida africana*, *Candida blankii*, and *Candida pulcherrima* are discussed in particular (Figure 1A,B and Figure 2). 

It is relevant to note that, although they are no longer considered as members of the genus *Candida* [8,9], the authors continued to address them here as *Candida*, as other reports have doing since the new names have been published. This will ease its recognition and the association between the infection and the case reports. The name of the new nomenclature will be displayed in parentheses in each section. The next sections describe the infections, treatment, and resistance reported for these *Candida* species.

### 2.1. Candida kefyr (New Nomenclature: Kluyveromyces marxianus or Candida pseudotropicalis)

*Candida kefyr* is an emerging and uncommon NCACs that is becoming more frequent over recent years. This fungal pathogen is diagnosed by histology, PCR, and DNA sequencing and has been reported in both superficial and systemic infections. Infections are of big concern in highly immunocompromised patients, and recent surveillance studies have reported that many isolates of *C. kefyr* are susceptible [20,21,22,23,24,25,26], but some have higher rates of resistance against established antifungals (amphotericin B, itraconazole, voriconazole, posaconazole, fluconazole, caspofungin micafungin, and anidulafungin) [27,28,29,30,31] (Table 1). A study at John’s Hopkins Hospital, USA, was performed, in which 83 patients were included and 8 patients (9.6%) were reported with *C. kefyr* colonization [29]. An unusual case of *C. kefyr* was reported in the bloodstream of an immunocompromised patient [30]. Biofilms of *C. kefyr* were reported in certain patient populations of Hungary, and amphotericin B, fluconazole, caspofungin, and micafungin were used for the treatment [31]. The treatment options for patients affected by these fungal infections have increased over the last few years; however, studies have also shown that delays (mostly due to the diagnosis and identification of *C. kefyr*) in the initiation of appropriate antifungal therapy ended in poor clinical compliance and outcomes [32]. This uncommon infection possesses additional diagnostic and therapeutic challenges [32]. *C. kefyr* has shown a particular high propensity (due to colonization) to cause disease and, therefore, resistance against the above-mentioned antifungals, worsening the treatment strategies [32]. Table 1 provides the compiled information of this species.

### 2.2. Candida norvegensis (New Nomenclature: Pichia norvegensis)

*Candida norvegensis* was identified in the sputum of three asthmatic patients in 1954, Norway [41], and the first case of a *C. norvegensis* peritonitis linked with peritoneal dialysis was reported in a renal transplant patient who developed *C. norvegensis* peritonitis [42]. Since then, only a few cases of *C. norvegensis* infection have been reported, mostly occurring in patients with cancer or HIV (Table 1) [43,44,45]. According to a recent study, the rate of *C. norvegensis* isolation grew by 5–10-fold in the previous decade [18]. The susceptibility of *C. norvegensis* to fluconazole and voriconazole showed that 41% of the isolates were resistant to fluconazole and 92% were sensitive to voriconazole, despite a recent increase in the number of voriconazole-resistant strains. Amphotericin B has long been regarded as the treatment of choice for *C. norvegensis* infections, even though the level of evidence is relatively poor due to the uncommon reports of infection [46]. The toxicity of amphotericin B, on the other hand, may limit its usage in solid organ transplant patients. Several reports have shown that *C. norvegensis* isolates are susceptible to echinocandins [47,48,49] (Table 2).

### 2.3. Candida inconspicua (New Nomenclature: Pichia cactophila)

The relative global incidence of *C. inconspicua* (formerly *Torulopsis inconspicua*) is rare but recurrent, and its prevalence has increased over 10-fold in the last few years [58]. Complications were also mostly reported from immunocompromised patients [59]. *C. inconspicua* was reported to be resistant to azoles but showed a susceptibility pattern with other antifungals, such as echinocandins and amphotericin B [54,59,60,61]. Due to the distinguished increase in reports of fluconazole resistance in this species of *Candida*, echinocandins are currently the first choice for the treatment of this infection (Table 3). Novel insights on the emergence, genetic diversity, infection pattern, molecular characteristics, and other associated information are needed for *C. inconspicua* [62].

### 2.4. Candida lipolytica (New Nomenclature: Yarrowia lipolytica)

*Candida lipolytica* can be found in the environment, as well in meat and dairy products, particularly cheese [63]. It is occasionally discovered as a colonizer, in asymptomatic people’s feces, oropharyngeal swabs, sputum, and skin swabs [64]. Aromatic compounds, organic acids, polyalcohols, emulsifiers, and surfactants are all produced by their strong secretory activity, which is widely used in the food, detergent, and pharmaceutical sectors [63,65,66]. Although *C. lipolytica* was once thought to have a low virulence, it is now widely recognized as a cause of sporadic cases and nosocomial clusters of human infections, particularly catheter-related suppurative thrombophlebitis and fungemia associated with biofilm formation in immunocompromised or critically ill patients who require long-term care (Table 4) [45,48,49]. Other clinical conditions have been documented, including non-catheter-related fungemia, traumatic eye infection, and the acute aggravation of chronic sinusitis [64,67]. Most of the research on *C. lipolytica* infections were case reports or short case series [64,67,68]. Trabelsi and colleagues detailed the epidemiological risk factors and clinical outcomes of 55 cases of *C. lipolytica* fungemia in Tunisia, as well as some information on the isolates’ in vitro sensitivity to a few antifungal medications [65]. However, in this large case series, data concerning the correlative microbiological features, such as phenotypic and genotypic identification, as well as in vitro susceptibility test findings for newer antifungal medications such as echinocandins and posaconazole, were absent. Recently, Zhao et al. described the epidemiological and clinical features of 13 cases of *C. lipolytica* fungemia in this multicenter, a prospective surveillance study in China, as well as the in vitro susceptibility of this emerging fungal pathogen to nine antifungal drugs, including the newer azoles and echinocandins [69]. In that study, the authors summarized that all isolates had low MICs to voriconazole, amphotericin B, and echinocandins but also demonstrated the need to establish standardized protocols to determine the in vitro antifungal susceptibility for *C. lipolytica* using different testing methods [69].

### 2.5. Candida lusitaniae (Updated Nomenclature: Clavispora lusitaniae)

*Candida lusitaniae* accounts for 0.2–9.4% of all *Candida* isolates from blood or other sterile places [79] and has been linked to amphotericin B resistance in the past (Table 5). Although there are no standardized protocols for assessing *Candida* susceptibility to amphotericin B, the current recommendations propose that any *Candida* species with an amphotericin B MIC > 1 g/mL be deemed resistant [80]. According to recent research [81], 98% of *C. lusitaniae* initial bloodstream isolates were amphotericin B (MIC 0.313–0.625 µg/mL)-sensitive, and 96% were fluconazole-susceptible. Another investigation found that clinical isolates collected before antifungal medication had significant frequencies of amphotericin B resistance [82]. There was no mention of the subsequent susceptibility patterns or clinical outcomes in any of these studies. Others have reported clinical failures associated with increases in amphotericin B MICs during amphotericin B treatment [81,83,84], and *C. lusitaniae* is known to switch between amphotericin B susceptibility and resistance in vitro [85]. Sometimes, even an extremely high concentration of amphotericin B (1 g/mL) failed to treat *C. lusitaniae* fungemia [83]. It can be speculated that this resistance might be related to mutations in the ergosterol biosynthetic pathway, with direct effects on gene expression. Then, a selective gene expression in the adaptive response to amphotericin B may occur, with high-frequency reversible phenotypic switching from susceptibility to resistance associated with distinct morphologies, as it happens with other *Candida* spp. [86].

### 2.6. Candida famata (New Nomenclature: Debaryomyces hansenii)

Very rare cases of candidemia have been reported with *Candida famata* (Table 6). They are found in food, marine, and terrestrial environments and are being recognized as potential emerging pathogens that cause human candidiasis. A 16-year old patient with Hodgkin’s disease undergoing chemotherapy was reported with this fungus. The patient received voriconazole for 3 weeks and improved [94]. Another study involved eight *C. famata* samples that showed resistance to fluconazole but susceptibility to posaconazole and caspofungin [95]. In another report involving two hospitalized patients with central venous catheters, *C. famata* isolates reduced their susceptibility to azoles and echinocandins. The patients were treated and cured with liposomal amphotericin B therapy [96]. Septic shock has been reported in healthy young multi-traumatic immunocompetent male patients due to this yeast. After the initiation of amphotericin B, the patient quickly recovered from sepsis and was discharged from the hospital [97]. These few case reports summarized the routine antifungal susceptibility testing in patients with candidemia to guide the optimal antifungal therapy.

### 2.7. Candida guilliermondii (New Nomenclature: Meyerozyma guilliermondii)

*Candida guilliermondii* is a rarely isolated and reported fungal pathogen from a clinical specimen. A very low mortality rate was associated with this species; however, it is known to be an opportunistic emerging pathogen causing candidiasis often associated with cancer patients [98]. The *C. guilliermondii* complex has been described by high antifungal resistance to fluconazole and echinocandins in 22 patients with *Candida* infection (*C. guilliermondii* (*n* = 17) and *C. fermentati* (*n* = 5)) [98]. In 1985, Dick et al. reported a case of a 52-year-old woman with candidemia due to *C. guilliermondii* [99]. The patient died regardless of amphotericin B therapy [99]. Fifty-two patients with infections from the *C. guilliermondii* complex (*C. guilliermondii* (n = 77) and *C. fermentati* (*n* = 5)) were studied by Chen et al. [100]. In this study, 98%, 100%, and 98% of *C. guilliermondii* isolates were susceptible to caspofungin, micafungin, and anidulafungin, respectively. There was a good in vitro activity of the above-mentioned antifungals against *C. guilliermondii* complex isolates (Table 6) [101].

**Table 6:** General information and characteristics of candidiasis described for *Candida famata* and *Candida guilliermondii*.

Invasive/NonInvasive Candidiasis (n Human Cases/Strains/Isolates)	Identification Methods	Imaging Test	Antifungal Susceptibility	Antifungal Resistance	Antifungal Treatment	Other Treatments (e.g., Probiotics, Natural Compounds, Antivirals)	Outcome (n)	Reference(s)
***Candida famata***								
(invasive) *C. famata* (n = 1)	Hodgkin’s disease, blood culture	Microscopy	Voriconazole	NR	voriconazole	NA	Alive	[94]
(invasive) *C. famata* (n = 8)	Urine culture	Microscopy	Posaconazole, caspofungin	Fluconazole	Posaconazole	NA	No death	[95]
(invasive) *C. famata* (n = 2)	Blood culture	Microscopy	Reduced to echinocandins, azoles	Azole resistance reported	Liposomal amphotericin B	NA	No death	[96]
(invasive) *C. famata* (n = 1)	Blood cultures	Partial amplification and sequencing of the 26S ribosomal DNA gene	Anidulafungin and micafungin	Fluconazole	Amphotericin B	NA	Alive	[97]
***Candida guilliermondii***								
(non-invasive) *C. guilliermondii* (n = 17)	Blood sample	Amplification and sequencing of the ITS1-5.8S-ITS2 region	NR	Fluconazole and echinocandins	Amphotericin B	NA	NR	[98]
(invasive) *C. guilliermondii* (n = 1)	Blood cultures	Microscopy	NR	Fluconazole	Patient died despite of amphotericin B therapy	NA	Died despite of amphotericin B therapy	[99]
(invasive) *C. guilliermondii* (n = 47)	Blood cultures	PCR- restriction fragment length polymorphism	Caspofungin, micafungin and anidulafungin	NR	NR	NA	No	[100]

MIC: Minimal inhibitory concentrations; NA: Not applicable, because the research is performed on fungal strains/clinical isolates; NR: Not reported; MALDI-TOF MS: matrix-assisted laser desorption ionization mass spectrometry [98,99,100,101].

### 2.8. Candida rugosa (New Nomenclature: Diutina rugosa)

*Candida rugosa* has lately been highlighted as one of the emerging fungal pathogens [101] and a source of invasive fungal diseases [102]. Before 1985, when catheter-related fungemia was documented at two distinct institutions in the United States, fungemia caused by *C. rugosa* remained unknown [103,104]. Thereafter, Dube et al. [105] reported 15 cases of candidemia caused by *C. rugosa* in burned patients treated with topical nystatin in a U.S. hospital. The infections had no evident cause, but the isolates were determined to be resistant to nystatin and had a reduced sensitivity to amphotericin B and fluconazole [105]. A cluster of six occurrences of candidemia caused by *C. rugosa* has recently been described in Brazil [106]. Two of the cases included breakthrough infections in patients being treated with amphotericin B, and all four patients who were given this medication died [106]. *C. rugosa* was a common colonizer of high-risk patients, accounting for 44% of 32 consecutive episodes of fungemia at one Brazilian tertiary care hospital, according to the follow-up monitoring study [107] (Table 7). These data suggest that *C. rugosa* can lead to catheter-related fungemia in critically ill patients. It can be transmitted from patient to patient in hospitals and be endemic in some institutions. It may also be resistant to polyenes and fluconazole [101]. In addition to these observations, nothing much is known about the epidemiology, frequency, and antifungal susceptibility profile of this rare *Candida* species. [102].

### 2.9. Candida pararugosa (New Numenclature: Wickerhamiella pararugosa)

*Candida pararugosa* is an emerging and rare yeast pathogen reported in both humans and animals in different organs and biological liquids (Table 2) [112]. Initially, in 1998, it was reported from human feces and then isolated from the oral cavity, where it was thought to illustrate colonization rather than a true infection [113]. The yeast was isolated from two different blood cultures in a 39-year-old-woman who developed post-abdominal surgery sepsis and surgical wound necrotizing fasciitis. Treatment with micafungin improved the patient’s clinical signs; however, it was not clear that sepsis resulted from candidemia or necrotizing fasciitis [113]. Concerning the literature, *C. pararugosa* seems to mainly leads to invasive fungal infection (IFI), predominantly in children and adults [112,114]. 

Candidemia caused by *C. pararugosa* is associated with a high morbidity and mortality, especially among immunocompromised patients [115]. Therefore, the precise identification of causative agents of the bloodstreams of particularly uncommon *Candida* species has paid high attention recently because of changing the epidemiology of candidemia. Moreover, clinical laboratories should be aware to identify the rare yeasts in specimens accurately for a faster treatment [34,116].

There are few available data for *C. pararugosa* infections regarding its identification, antifungal susceptibility testing, clinical significance, and treatment protocols [113]; however, the number of reports related to different aspects of *C. pararugosa* has recently risen worldwide, as we are witnessing an increase in the prevalence of *C. pararugosa*. In a case report by Piatti et al., *C. pararugosa* was isolated from the blood culture of a 65-year-old woman diagnosed with metastatic lobular breast cancer carrying a central venous catheter that was under a chemotherapy regime [112]. Interestingly, an elevated level of glucan was also detected in the blood analysis. Based on the antifungal susceptibility testing results, *C. pararugosa* showed a lower MIC against fluconazole. Hence, she underwent fluconazole therapy (initially with a load dose, 800 mg orally, on day 1 and then with 400 mg daily for two weeks). Nonetheless, the patient died due to malignancy [112].

A retrospective study was carried out from 2008 to 2020 in the largest tertiary Greek pediatric hospital. Fourteen different rare fungal species in 33 neonates and children with IFI hospitalized in the intensive care (ICU) and oncology units isolated from central catheters, peritoneal, pleural, blood, and pericardial fluid specimens were involved. *C. pararugosa* was identified using conventional, molecular, and MALDI–TOF MS methods, and the antifungal susceptibility profile was performed according to CLSI. Disappointedly, no official antifungal breakpoints have been defined for these rare yeasts [114]. Stavrou et al. reported that clinical isolates of *C. pararugosa* elevated MICs against common antifungal drugs (according to the EUCAST broth microdilution method). Amphotericin B was the most efficient drug, whereas azoles and echinocandins had high MICs. Surprisingly, voriconazole had the broadest efficacy among azole drugs [50]. Similarly, in another study in 2019, the identification of rare yeast isolates of clinical origin was attained by MALDI-TOF MS or internal transcribed spacer sequencing. The antifungal susceptibility patterns were generated for azoles, echinocandins, and amphotericin B using the commercial E-test and the EUCAST broth microdilution method. The findings showed that *C. pararugosa* had elevated echinocandin MICs (MIC50 ≥ 0.06 mg/L) and shared high fluconazole MICs, suggesting that the MIC values generated with E-test cannot be directly compared with the EUCAST results [61]. According to a report at a tertiary teaching hospital in Malaysia*,* seven isolates of the *C. rugosa* complex and one isolate of *C. pararugosa* were included to determine the antifungal susceptibility testing, biofilm formation, and enzyme activity of isolates. The E-test showed that both species have elevated MICs compared to *C. albicans* and *C. tropicalis*. All isolates exhibited high proteinase activity with a high capacity for biofilm formation, while none of the isolates exhibited phospholipase activity [117]. In a retrospective study in Italy, 156 yeast isolates were collected during 17 months from clinical samples of the microbiology department; approximately 2.1% of isolates were identified as *C. pararugosa* using MALDI-TOF MS [34]. The increase in the prevalence of the NCACs throughout the years was reported in Brazil (2007–2010) [34,116]. Yeasts from the blood of 104 patients were isolated, and the *Candida* spp. Were characterized by phenotypic and genotypic methods, and *C. pararugosa* was detected in one of these cases [34,116]. In Qatar, pediatric and elderly patients with fungemia have also been positive for *C. pararugosa*. MALDI-TOF MS provided the correct identifications compared with molecular analysis testing of the same isolates. All yeasts showed low MICs against isavuconazole and voriconazole as well [78].

The importance of accurate identification of the *C. rugosa* complex is much clearer when a molecular analysis of the sequences of the D1/D2 domains and the internal transcribed spacer (ITS) region of the ribosomal genes were used. In this context, a study by Parades et al. to distinguish 24 clinical isolates that phenotypically identified as *C. rugosa* revealed that only 10 (41.6%) isolates belong to that species, and the rest of the isolates identified as *C. pararugosa* and *C. pseudorugosa* and *C. neorugosa*, respectively, based on the similar D1/D2 sequences [118].

### 2.10. Candida pelliculosa (New Nomenclature: Wickerhamomyces anomalus)

*Candida pelliculosa* (formerly *Pichia anomala* or *Hansenula anomala)* is an ecological fungal species causing infections in immunocompromised hosts. Surprisingly, it has been proposed for many biotechnological applications in the food industry [119]. Feed and food supplemented with certain *C. pelliculosa* strains show an improved quality due, for example, to the addition of advantageous proteins and phytase activity [120]. 

*C. pelliculosa* was isolated from pigeons and their droppings as reservoirs and carriers of yeast that affect public health [121]. Only a few cases in the literature globally have illustrated that *C. pelliculosa* infects patients, particularly neonates hospitalized in the neonatal intensive care unit resulting in outbreaks of neonatal candidemia [122,123,124,125]. It has been reported that the common symptoms of *C. pelliculosa* candidemia were fever, cyanosis, polypnea, hypoactivity, and apnea. Mostly, as other *Candida* spp., immunosuppressed individuals using broad-spectrum antimicrobials or a long-term stay at the hospital are probably linked to the risk of infection with *C. pelliculosa* [126]. Isolates from neonates diagnosed with candidemia caused by *C. pelliculosa* in China showed high susceptibility to the antifungal activity of fluconazole, voriconazole, amphotericin B, and 5-fluorocytosine, which were from two different clones of *C. pelliculosa* [127]. It is recommended that monitoring rare strains isolated from immunodeficient hosts is critical to prevent possible outbreaks and control hospital-acquired infections, due to the clinical signs of disease nonspecific in the patients [127]. *C. pelliculosa, Candida utilis,* and *Candida fabianii* are hard to discriminate using common biochemical tests; however, an accurate identification of *C. pelliculosa* can be reached by biochemical kits, MALDI-TOF MS, and qPCR [119,128]. In 2015 was the first report of a nosocomial candidemia outbreak involving 11 patients in two ICUs and two general wards caused by *C. pelliculosa* in South Korea. The study showed that these isolates were similar in the randomly amplified polymorphic DNA (RAPD) assay [123]. Medical staff and staying in the interventional radiology procedure room were risk factors for the development of fungemia, and the outbreak was eradicated using strict hand washing, disinfecting medical equipment, and contact precautions [123]. 

Several findings demonstrated the antimicrobial potential activity and wide range of biotechnological characteristics of *C. pelliculosa* that received considerable attention [120]. Anti-idiotypic antibodies generating an “internal image” of a killer protein have been found to possess therapeutic activity against a broad range of microorganisms [120]. Particularly, a purified protein with a molecular weight of 140 kDa was isolated from a specific strain of *C. pelliculosa* isolated from *Anopheles stephensi*—namely, WaF17.12—corresponding to a high molecular weight, β-glucosidase produced a killer toxin with strong anti-plasmodial activity [129]. Consistently, the production of glucanases coded by the “killer genes” WaEXG1 and WaEXG2 of *C. pelliculosa* has been investigated as a biocontrol agent to play a role in the ability of yeast to inhibit other fungi [130]. Additionally, the antifungal activities of new N-donor bitriazolic tripods were reported against the fungal strain *C. pelliculosa*. Molecular docking studies of some compounds indicated that they could act as inhibitors for the biotin carboxylase enzyme [131]. In a study by Paris et al., the mycocin activity was obtained from the cell wall of environmental *C. pelliculosa* (WA40, WA45, and WA92), which exhibited antifungal activity against thirty *C. albicans* strains from candidemia [132]. Similarly, another mycotoxin was isolated from the culture supernatant of *C. pelliculosa*—exo-β-1,3 glucanase—characterized by MALDI-TOF MS, which had antifungal activity against *Candida mesorugosa* but not against *C. albicans*, *C. parapsilosis*, and *C. krusei* [133]. Moreover, secondary metabolites by *Streptomyces* spp. TUR-10, indicated antifungal activity against the clinical isolate of *C. pelliculosa* with significant antifungal activity values ranging from 15.6 to 250 μg/mL, suggesting abundant potential for further research [134]. Some cases have been reported to be associated with *C. pelliculosa* infections in patients globally, which is shown in Table 8.

### 2.11. Candida nivariensis (New Nomenclature: Nakaseomyces nivariensisa)

*Candida nivariensis* is closely related to the *Candida glabrata* complex and has become a global increasingly emerging and cryptic fungal species. It was described for the first time in 2005 by DNA sequencing [140]. Little is known regarding *C. nivariensis* and *Candida bracarensis*. Although there have been efforts to isolate *C. nivariensis* from large samples of the *C. glabrata* complex, these species have not been identified in some countries [141]. Nonetheless, several patients receiving fluconazole (treatment or prophylaxis) for *C. nivariensis* have been experiencing an increased rate of therapeutic failure in the UK. Therefore, a rapid and efficient yeast identification would be clinically important to decide whether fluconazole would be a suitable treatment for members of the *Nakaseomyces* clade, especially *C. nivariensis* [142]. Although *Candida glabrata* complex species are difficult to identify by traditional laboratory methods, there are cost-effective methods that can properly identify different species [141,143,144,145]. To date, MALDI-TOF MS is sensitive and has a practical applicability in the rapid detection of the *C. glabrata* species complex, indicating promising results for such a purpose [140]. The high-resolution melting curve (HRM) method targeted the ITS region to design a specific primer of clinical isolates that consists of DNA sequencing. The method allows for the early and targeted treatment of patients with invasive candidiasis [146]. 

Compared to *C. albicans*, *C. nivariensis* is more virulent and resistant to antifungal drugs [147]. The efficacy of the most common drugs against *C*. *nivariensis* isolates was evaluated in an in vivo model, *Caenorhabditis elegans*. The results showed that echinocandins had a higher efficacy to treat *C. nivariensis* infections [148]. The available data on antifungal susceptibility profiles of the *C. glabrata* complex are still very few [149]; yet, some reports are accessible. The first case of candidemia was reported in an 81-year-old man who was hospitalized for the surgical treatment of intestinal fistula caused by *C. nivariensis* (associated with a catheter). Due to a failure treatment with fluconazole and to a positive blood culture during treatment, the antifungal treatment was successfully changed to intravenous caspofungin for 2 weeks [150]. In another report (Poland), 24 *C. nivariensis* isolates were isolated from 445 clinical samples. Ninety-two percent of *C. nivariensis* were resistant to itraconazole, and half were resistant to posaconazole. Eighty-three percent of *C. nivariensis* were susceptible to voriconazole, while all strains were fluconazole-resistant. This clearly indicates that *C. nivariensis* should be considered as an emerging pathogen, with a relevant resistance to azoles [151]. Shi et al. evaluated several MIC geometric means of antifungal drugs (e.g., caspofungin, fluconazole, itraconazole, and amphotericin B) in women with vulvovaginal candidiasis (VVC). The authors showed that the *C. nivariensis* isolates were higher than those in *C. albicans* and that the level of resistant genes *ERG11, CDR1,* and *CDR2* and virulent genes *YPS1*, *AWP3*, and *EPA1* mRNA expression were higher in *C. nivariensis* isolates compared to *C. glabrata*, which is clinically serious [147]. In another study, all *C. nivariensis* isolates were susceptible to nystatin and susceptible or susceptible dose-dependently to fluconazole, itraconazole, miconazole, and clotrimazole. Importantly, the therapeutic efficacy in the patients was poor and inconsistent with the observed in vitro antifungal susceptibility; thus, extra studies are required [152]. Moreover, four Delhi patients with VVC related to *C. nivariensis* were resistant to fluconazole but susceptible to voriconazole, itraconazole, posaconazole, isavuconazole, amphotericin B, and echinocandins Similarly, in Iran, 4 out of 213 clinical *C. glabrata* species complex isolated from candidemia cases and identified as *C. nivariensis* were susceptible to amphotericin B, fluconazole, itraconazole, posaconazole, voriconazole, anidulafungin, and micafungin [143]. Not long ago, the antifungal susceptibility profile of 122 *C. glabrata* complex strains (including 5 *C. nivariensis* and 3 *C. bracarensis* strains) were evaluated and compared with the findings of the *FKS* gene mutations. Except for one isolate, no echinocandin resistance was detected, which was consistent with the MIC results. *FKS* sequencing results of the *C. glabrata* isolates were different from *C. nivariensis* [153]. Biofilm studies involving *C. nivariensis* have also been reported. The biofilm formation and antifungal susceptibility profile were evaluated in a clinical strain of *C. nivariensis* compared with the standard strains for the first time in Brazil. All strains showed low planktonic MICs to amphotericin B, caspofungin, and voriconazole while resistant to fluconazole. However, increasing the planktonic MICs to Posaconazole and itraconazole, the isolates produced a high level of protease enzyme as a virulence factor [144]. Finally, a *C. elegans* model was used for the simulation of *C. glabrata* and *C. bracarensis* infection. The results demonstrated an easy eradication of the infection by amphotericin B and azoles, while echinocandins were more effective against *C. nivariensis* [148,154].

### 2.12. Candida bracarensis (New Nomenclature: Nakaseomyces bracarensisa)

Fifteen years ago, during *Candida* species epidemiological research that took place in Braga (Portugal), the new name “*Candida bracarensis*” was given to a strain phylogenetically close to *Candida glabrata* [155,156]. Due to developments in molecular methods and the results of detailed analyses, *C. glabrata* was introduced as a complex of *C. glabrata, C. nivariensis*, and *C. bracarensis* [146,148,157]. In fact, the accurate identification of ambiguous species such as *C. nivariensis* and *C. bracarensis* is important, but in several geographical areas, these species remain unclear [148], and, currently, there is still a lack of information about *C. bracarensis* epidemiology or virulence factors [154]. Reports have described *C. bracarensis*, together with *C. nivariensis*, as etiological agents of VVC [152], since some strains have been isolated from a vaginal swab [154] or from samples of symptomatic pregnant women [152]. Since then, vaginal presumptive *C. glabrata* isolates were also retrospectively rechecked for *C. nivariensis* and *C. bracarensis* [152]. The data about clinical therapeutic efficacy and the in vitro antifungal susceptibility of *C. bracarensis* is still poor [152], but it seems that *C. bracarensis* has a slightly distinct phenotype and antifungal susceptibility profile from *C. glabrata* [158]. Obviously, an early, fast, and exact identification system that distinguishes these three species is crucial for targeted medication [146,158] Nowadays, reexamination is performed for the control of previously identified strains. In fact, numerous isolates of *C. glabrata* that have been reassessed, *C. glabrata sensu stricto* was confirmed for all of them [159]. The first identification of the phenotypes is commonly realized by cultivation using CHROMagar *Candida* medium [160], but another option is identified by the API^®^ *Candida* system [152]. The results of traditional laboratory methods can be supported by denaturing high-performance liquid chromatography (dHPLC), which is fast and provides an up-to-date multiple analysis of *Candida* species in various samples [158]. Furthermore, as previously explained, molecular diagnostic methods have proven to be highly efficient in the correct identification of pathogenic yeasts, including *C. bracarensisis* and *C. nivariensis* (e.g., DNA sequencing is highly specific, using the rDNA ITS region, which offers an accurate diagnostic and can be applied as a reference tool [161], ITS2-MCA [145], HRM evaluation [146], mPCR, and three species-specific single-plex PCR [161,162]). In Spain, sequencing analysis indicated that 3 of 143 isolates (2%) were *C. bracarensis* [161]. Over 300 *C. glabrata* isolates from children and adults were initially biochemically distinguished via multiplex PCR, sequencing, and MALDI-TOF MS. One strain was found to be *C. bracarensis* [161]. Vitek MS^®^ Research Use Only system and Bruker ClinProTools software proved 100% capable of discrimination and cross-validation for *C. bracarensis* and *C. nivariensis* [163]. Another retrospective re-examination of the vaginal *C. glabrata* samples via the ITS1 region, and the 5.8S ribosomal RNA gene assays pointed out that 293 in 301 isolates were correctly identified (*C. glabrata*). By sequencing, it was confirmed that the remaining isolates were *C. nivariensis* (7) and one as *C. bracarensis* [152]. In another study related to isolates of the *C. glabrata* complex, none of the *C. bracarensis* strains were found (via sequencing the D1/D2 region of 26S rRNA)[160]. It is also relevant to note that a single primer pair targeting the RPL31 gene (a gene coding for a protein component of the large ribosomal subunit) can also be used as a potential tool to distinguish between *C. glabrata*, *C. bracarensis*, and *C. nivariensis* [144].

Presently, there are only a few reports about the antifungal susceptibility pattern of *C. bracarensis* [159], but in candidiasis, generally, the most often selected triazole for patients is fluconazole, both for the therapy of candidiasis but also for prophylaxis [142]. *C. bracarensis* is also found in Mexico. This isolate was determined as susceptible to echinocandins (caspofungin, anidulafungin, and micafungin; MIC = 0.031 μg/mL). Interestingly, the authors also observed a noticeable activity of aspartyl proteinase, phospholipase, and hemolysin in this strain [144,156]. An extensive 15-year survey concerning 82 species of uncommonly occurring yeasts and yeast-like fungi (e.g., members of the *Nakaseomyces* clade: *C. glabrata*, *C. nivariensis*, and *C. bracarensis*) showed no antifungal resistance patterns of *C. bracarensis* samples. The drugs included amphotericin B, fluconazole, itraconazole, voriconazole, posaconazole, and anidulafungin [142]. Another research concluded that azoles (e.g., fluconazole, itraconazole, miconazole, and clotrimazole) and nystatin (a polyene) were effective against all tested *C. bracarensis* and *C. nivariensis* isolates [152].Using conventional mycological methods in oral samples, *C. glabrata*; *C. parapsilosis*; and their cryptic species: *C. bracarensis*, *C. nivariensis*, *C. metapsilosis,* and *C. orthopsilosis* were detected and identification was confirmed by molecular assays. Disk diffusion and microdilution results of the in vitro susceptibility assay showed the efficacy of miconazole and nystatin against most *C. glabrata* isolates, but they were resistant to fluconazole and itraconazole [156]. The *FKS* genes analysis of mutations (for echinocandins resistance) did not show any evidence of echinocandins resistance in *C. glabrata* complex strains (five *C. nivariensis* and three *C. bracarensis)* [164]. Finally, the ability to form a biofilm of *C. bracarensis* strains was described as an important virulence factor [156]: while planktonic cells are susceptible to antifungals, amphotericin B or fluconazole were not able to stop biofilm development [156]. Table 9 summarizes the information on *C. bracarensis*-reported cases.

### 2.13. Candida intermedia

*Candida intermedia* has been mainly reported in cases related to bloodstream infections in Asia [165]. The first case was confirmed using the molecular method (D1/D2 domain of the large subunit 26S rRNA gene), but there was an initial misidentification with the phenotype test that revealed a false result for *Cryptococcus humicola* [165]. In Iran, there is particular care on the surveillance of rarely occurring *Candida* species that cause candidemia in intensive care units, because candidiasis is still a serious problem in this country (Table 4). Indeed, *C. intermedia* belonged to this group of main pathogens, accounting for about 11% together with *C. orthopsilosis, C. glabrata, Candida dubliniensis, Candida lusitaniae,* and *Candida kefyr* [166]. In Qatar, *C. intermedia* has been identified in pediatric and elderly patient samples, using molecular identification and MALDI-TOF MS. The results of the antifungal susceptibility testing showed only rare cases of resistance, and isavuconazole and voriconazole successfully inhibited the tested species [78]. Furthermore, *C. intermedia* was one of the 82 vulvovaginal isolates verified by conventional mycological methods, with a high proteinase activity [167]. Curiously, it was also detected from the soil by the D1/D2 domain of the 26S rRNA gene amplification, sequence determination, and the phylogenetic analysis [168]. Table 9 has more information related to this species.

### 2.14. Candida africana

*Candida africana* is a newly described opportunistic yeast pathogen that is related to invasive and VVC [169,170] (Table 10). Based on biochemical, morphological, and physiological characteristics, this pathogen was first described, in 1995, as an atypical chlamydospore-negative *C. albicans* strain and subsequently proposed as a new *Candida* species that is different from typical *C. albicans* isolates [170]. Presently, the prevalence of *C. africana* species and its epidemiological assessment in clinical samples are still unknown, mostly because diagnostic laboratories use phenotypic identification systems that do not always allow discrimination between variants or closely related species of *Candida*. In fact, in a recent retrospective study with 52 culture collection isolates, two isolates were found to be *C. africana* using molecular methods [171]. However, initially, most of the isolates of *C. africana* were isolated from female genitalia [172], and vaginal tracts are frequently affected by this species [170,171,172,173,174]. One isolate of *C. africana* was recovered from a blood culture in Chile, South America, and it is also possible that *C. africana* may be associated with a wider clinical spectrum [174].

Due to *C. africana* infection, most candidiasis cases have been reported from seven countries of the African continent [169,175,176]. One hundred and fifteen (40.6%) patients with *C. africana* candidiasis belong to this region. Madagascar and Angola had the majority of the cases (*n* = 93, 80.8%). The tropical and subtropical climate and high temperatures in Angola and Madagascar may contribute to *C. africana* prevalence. However, 87.6% of the infections with this species have been reported from the genital specimens of South African patients, and the distribution of *C. africana* infections was relatively higher among age groups of 18–35 years compared to others. Due to a weakened immune system, frequent sexual activities, and the use of oral contraceptives, pregnancy should be considered more as the main reason associated with a more prone condition to *C. africana* vaginitis. The overall prevalence of *C. africana* vaginal infections (1.9%) was close to the reports (1.4%) in India [177], (0.4%) Turkey [170,178], and (0.3%) Saudi Arabia [170]. However, it was lower as compared to those reported in Iran (3.2%, 5%, and 8.4%) [179,180,181]; China (6.3%) [182]; and the UK (5.9%) [183]. Environmental factors and differences among the study participants, including non-pregnant and pregnant women, symptomatic and asymptomatic conditions, immunity, personal hygiene practice, and patients’ socioeconomic status might explain this inconsistency. Although antifungal susceptibility testing in vaginal isolates is recommended because vaginal candidiasis is one of the reasons for frequent antimycotic medication among women of reproductive ages., *C. africana* isolates remain susceptible to commonly used antifungal agents with increasing antifungal resistance [184]. In this study, the high susceptibility of *C. africana* was also observed against all tested antifungals, and a resistance to flucytosine, voriconazole, and terbinafine have been reported against *C. africana* isolates [170,184].

**Table 10:** General information and characteristics of the candidiasis described for *Candida africana*.

Invasive/Noninvasive Candidiasis (n Human Cases/Strains/Isolates)	Identification Methods	Imaging Test	Antifungal Susceptibility	Antifungal Resistance	Antifungal Treatment	Other Treatments (e.g., Probiotics, Natural Compounds, Antivirals)	Outcome (n)	Reference(s)
(invasive) *C. africana* (n = 2)	MALDI-TOF MS and PCR	Microscopy	Ketoconazole (one) Fluconazole (one) Itraconazole (both) Amphotericin B (one)	Ketoconazole (one) Fluconazole (one) Amphotericin B (one)	NR	NA	Alive	[185]
*Candida* vaginitis (n = 10)	PCR-RFLP and sequencing CHROMagar Candida	Microscopy	Fluconazole	NR	NR	NA	NA	[181]
(invasive) *C. africana* (n = 2)	CHROMagar and PCR	Microscopy	Amphotericin B, fluconazole, and itraconazole	Fluconazole and itraconazole	NR	NA	NA	[180]
(invasive) *C. africana* (n = 3)	CHROMagar MALDI-TOF MS	Microscopy	Fluconazole; voriconazole; ketoconazole; amphotericin B; anidulafungin; micafungin	NR	NR	NA	NA	[178]
(invasive) *C. africana* (n = 1)	CHROMagar and PCR	Microscopy	Amphotericin B, nystatin, fluconazole, itraconazole Voriconazole, clotrimazole, terbinafine	NR	NR	NA	NA	[186]
(invasive) *C. africana* (n = 5)	CHROMagar and PCR	Microscopy	Caspofungin, anidulafungin, micafungin, itraconazole, voriconazole, posaconazole	NR	NR	NA	All alive	[179]
(invasive) *C. africana* (n = 15)	CHROMagar and PCR	Microscopy	Fluconazole, itraconazole, miconazole, clotrimazole,	NR	NR	NA	All alive	[187]
(invasive) *C. africana* (n = 4)	CHROMagar and PCR sequencing	Microscopy	Nystatin, clotrimazole, isavuconazole, ketoconazole, miconazole and posaconazole	NR	NR	NA	All alive	[177]
(invasive) *C. africana* (n = 15)	CHROMagar and PCR sequencing	Microscopy	Amphotericin B, nystatin, Itraconazole, miconazole, econazole, and ketoconazole	NR		NA	Alive (14) Dead (1)	[183]
(invasive) *C. africana* (n = 2)	CHROMagar and PCR	Microscopy	amphotericin B, 5-fluorocytosine, Fluconazole, itraconazole, ketoconazole, voriconazole, posaconazole and caspofungin	NR	NR	NA	Alive	[188]
(invasive) *C. africana* (n = 1)	CHROMagar and PCR	Microscopy	Amphotericin B, 5-fluorocytosine fluconazole itraconazole, ketoconazole voriconazole	NR	NR	NA	Alive	[173]

MALDI-TOF MS: matrix-assisted laser desorption ionization mass spectroscopy; PCR: polymerase chain reaction; NR: not reported; NA: nonapplicable.

### 2.15. Candida blankii

In 1968, Buckley and van Uden described a newly discovered nonfermenting yeast—*C. blankii*—from an infected mink in Canada, and the name was given in honor of Dr. Blank, who identified it [189]. The species was considered as nonpathogenic until 2015. Thereafter, it was isolated from the airways of patients with cystic fibrosis and reported to cause bloodstream infections (Table 11) [190]. Chowdhary and colleagues (2020) identified an outbreak of nosocomial fungemia caused by multidrug-resistant *C. blankii* (nine positive blood samples) in neonatal care in India for 7 months. Four of the neonates died. Importantly, the average MICs of two different antifungal drugs: fluconazole (8 mg/L) and anidulafungin (2 mg/L) were increased, and the genome sequencing results showed high probable antifungal resistance development [191]. Another study reported the death of a preterm neonate with a confirmed bloodstream infection due to *C. blankii* (PCR sequencing of rDNA), receiving combination antifungal therapy with amphotericin B and caspofungin [192]. In 2021, a case report concerning an adult immunocompromised patient with *C. blankii* endocarditis was published for the first time. Although there are still no official guidelines for the treatment of candidiasis caused by this opportunistic pathogen, the treatment was successful using a combination of polyene and echinocandins [193]. The assesment of marine fungal diversity on the Vietnam coast, by ITS sequencing, revealed an ecosystem composed of over 70 fungal isolates. Surprisingly, *C. blankii* was the most abundant species with 15 strains, originating from all surface coastal marine habitats, except two beaches [194].

### 2.16. Candida pulcherrima (Updated Nomenclature: Metschnikowia pulcherrima)

In 2012, *C. pulcherrima* was reported by Berkenzi et al. for the first time in a premature newborn girl in an invasive fungemia due to a catheter (parenteral nutrition). The strain was identified by microbiological and molecular assay (e.g., sequencing of a fragment of internal transcribed spacer ITS 1 and ITS 4 regions). The patient was treated by amphotericin B lipid complex therapy (5 mg/kg/d), since, initially, fluconazole therapy was not successful [195]. Similarly, a case report (Greece, 2016) of a neonate with prematurity and respiratory distress syndrome indicated fungemia because of *C. pulcherrima*, successfully treated with a combination of liposomal amphotericin B and micafungin [196]. The first case of a community acquired candidemia by *C. pulcherrima* was detected in a 48-year-old man in 2016. The final identification was carried out using MALDI-TOF MS, and contrary to the hospital isolates (abovementioned studies), the patient underwent fluconazole therapy with a satisfied outcome [197]. On the other hand, in a respective study between 2012 and 2017, 50 hospitalized patients with different predisposing factors were enrolled. From the *Candida* species isolation tests, *C. albicans* was dominant, followed by NCACs. Meaningfully, the *C. pulcherrima* recovered was from 2% of the patients. The highest antifungal sensitivity rates (>90%) were measured for amphotericin B, voriconazole, and echinocandins (Table 12) [198]. In another respective study involving 107 hospitalized patients with candidemia, 109 *Candida* species were identified. *C. albicans* was the most isolated, followed by NCACs: *C. parapsilosis* complex, *C. glabrata* complex, *C. tropicalis*, *C. krusei, C. lipolytica*, *Candida membranaefaciens*, and one *Candida pulcherrima*. The major risk factor was, in both adults and children, catheter use. Additionally, 8.5% of those NCACs were resistant to fluconazole [199].

Curiously, the antimicrobial activity of a new strain of *C. pulcherrima* was examined against fungal and bacterial species. Strong antagonistic activities of the *C. pulcherrima* strain were detected on the human pathogens *Escherichia coli*, *C. albicans*, *C. parapsilosis*, *C. krusei*, and *Trichosporon mucoides*. This was directly perceived by the same amount of the pigment pulcherrimin with different levels of antimicrobial effects on various species [200].

## 3. Conclusions

In the last decade, atypical *Candida* spp. have been related to several human fungal infections, from superficial to systemic diseases. The key case reports show that these yeast pathogens have, in some cases, a noteworthy profile of resistance to antifungal drugs, especially to azoles; however, resistance to polyenes and echinocandins has also been increasing. 

Pointedly, the absence of official clinical breakpoints and antifungal susceptibility profiles for most of the uncommon *Candida* spp. cited here makes the interpretation and treatment options a clinical challenge. In fact, one of the major problems that the scientific and clinical community faces with uncommon *Candida* spp. is that there is no official information on antifungal drug MIC values (both EUCAST and CLSI), and even the great majority of the analyzed reports do not present these values. Unsurprisingly, this makes it difficult to evaluate the antifungal profile and, therefore, to guide the treatment of fungal diseases, in this case, of *Candida* spp. infection. Additionally, defining “sensible/tolerant/resistant” inference is done by comparing MIC values with *Candida* common species (which already have official clinical breakpoints). This, of course, brings up several drawbacks in terms of the effectiveness of the antifungal therapy applied in these cases.

It is also important to note that there is a limitation in our work: in some countries, certain species (such as *Candida* uncommon species) are only used in research and not in clinical data (e.g., no final identifications, misidentification, and no/few/inadequate equipment/methods available). Obviously, an overview of the problem in terms of the evaluation of the epidemiology scenario is difficult, because, in these cases, there are no reports or because the available reports mislead us to common species that are, in fact, misidentified. Taken together, since the emerging and cryptic clinically important species are augmented, periodic surveillance, including the use of molecular identification methods, seems to be necessary for enlightening their frequency, geographical distribution, and susceptibility profile [201].

Due to the above, the scientific and clinical community must make efforts to publish breakpoints for a far higher range of *Candida* spp., increasing and focusing research on novel quick methods of diagnostic and new treatment strategies, to be able to fight these challenging infections in a timely manner. 

## Figures and Tables

**Table 1:** General information and characteristics of the candidiasis described for *Candida kefyr*.

Invasive/Noninvasive Candidiasis (n Human Cases/Strains/Isolates)	Identification Methods	Imaging Test	Antifungal Susceptibility	Antifungal Resistance	Antifungal Treatment	Other Treatments (e.g., Probiotics, Natural Compounds, Antivirals)	Outcome (n)	Reference(s)
(noninvasive) Cutaneous candidiasis (n = 1)	Carbohydrate fermentation test	Microscopy	Yes	NR	Miconazole cream and fusidic cream	Tenofovir, lamivudine, and efavirenz	Alive (n = 1)	[22]
(invasive) Bloodstream infection (n = 1)	Culture	Microscopy	No at low dose (100-200 mg fluconazole)	Yes	Fluconazole (high and prolonged dose)	NR	Successfully treated with 3 months use of fluconazole (n = 1)	[30]
(noninvasive) Infection at the interface of graft and host cornea (n = 1)	MIC	Optical coherence tomography	Yes	No	Fluconazole, voriconazole	Dexamethasone	Alive (n = 1)	[33]
(invasive) Infection at blood, respiratory, and urine samples (n = 69)	PCR, antifungal susceptibility testing	NR	Some strains were susceptible	Fluconazole and voriconazole, caspofungin and micafungin, amphotericin B	Fluconazole, voriconazole, caspofungin, micafungin, amphotericin B	NR	NA	[27]
(invasive) Bloodstream infection (n = 1)	MIC, genome sequencing	MRI	Yes	NR	Fluconazole, posaconazole	Trimethoprim, sulfamethoxazole	Alive (n = 1)	[25]
(invasive) Tubo-ovarian abscess (n = 1)	Culture	CT scan	Yes	No	Fluconazole	Consumption of organic dairy products	Alive (n = 1)	[23]
(invasive and noninvasive) Superficial and/or invasive infections (n = 2)	Anti-fungal susceptibility testing, Mass spectrometry	NR	Yes	No	Amphotericin B, itraconazole, voriconazole, posaconazole, fluconazole, caspofungin micafungin, anidulafungin	NR	NA	[34]
(invasive) Derived from blood, urine, bronchus, abdominal, and throat samples (n = 10)	Antifungal susceptibility testing,	Scanning electron microscopy	Lower in vitro susceptibility	Development of resistance	Fluconazole, amphotericin B, caspofungin, micafungin	No	NA	[31]
(noninvasive) Mucocutaneous candidiasis (n = 10)	Antifungal susceptibility testing, PCR, Sequencing	NR	Yes	No	Fluconazole, itraconazole amphotericin B	No	NA	[24]
(invasive) Bloodstream infection (n = 3)	Biochemical and molecular methods	NR	Susceptible to most of the antifungals	Amphotericin B	Fluconazole, voriconazole, caspo/anidulafungin, amphotericin B	No	NA	[28]
(invasive) Bloodstream infection (n = 1)	PCR, sequencing of the ITS region ofrDNA	NR	Yes	No	Amphotericin B, itraconazole, voriconazole, posaconazole, fluconazole, caspofungin micafungin, anidulafungin	No	NA	[21]
(invasive) Bloodstream infection (n = 2)	Gram staining, and germ tube test	Microscopy	Yes	No	Azoles, echinocandins	No	NA	[20]
(invasive) (n = 83)	Antifungal susceptibility testing, PCR	NR	No	Yes	Micafungin, liposomal amphotericin B, flucytosine	Yogurt	All alive (n = 83)	[29]
(invasive) Fungal sinusitis (n = 1)	Germ tube and sugar assimilation test	NR	Yes	No	Amphotericin B	No	Recovered completely (n = 1)	[35]
(invasive) Blood, bile and stool infection (n = 1)	Antifungal susceptibility testing, PCR	NR	No	Yes	Caspofungin, micafungin, and anidulafungin	No	Recovered completely (n = 1)	[36]
*Saliva* (n = 92)	Antifungal susceptibility testing, RAPD	NR	Yes	No	Fluconazole and itraconazole	No	Alive (n = 92)	[37]
(invasive and noninvasive) Blood, saliva, urine, broncho alveolar lavage (n = 410)	Germ tube and chlamydospore production tests	NR	Yes	Resistant to Itraconazole	Ketoconazole, itraconazole, voriconazole, caspofungin, amphotericin B	No	No death reported	[38]
(invasive) Systemic candidiasis (n = 1)	Ellipsometer test, PCR	NR	Yes	No	Liposomal amphotericin B, fluconazole	Broad spectrum antibiotics	Recovered completely (n = 1)	[39]
(invasive) Blood and urine samples (n = 3)	Culture, RAPD, ophthalmologic	Echocardiography, ultrasound check	Yes	No	Liposomal amphotericin B, fluconazole, and itraconazole	No	No death reported (n = 3)	[40]

MIC: Minimal inhibitory concentrations; NA: Not applicable, because the research is performed on fungal strains/clinical isolates; NR: Not reported; MALDI-TOF MS: matrix-assisted laser desorption ionization mass spectrometry.

**Table 2:** General information and characteristics of candidiasis described for *Candida norvegensis*.

Invasive/Noninvasive Candidiasis (n Human Cases/Strains/Isolates)	Identification Methods	Imaging Test	Antifungal Susceptibility	Antifungal Resistance	Antifungal Treatment	Other Treatments (e.g., Probiotics, Natural Compounds, Antivirals)	Outcome (n)	Reference(s)
(invasive) Clinical isolates (n = 14)	MIC, Fungicidal and fungistatic activity	NR	Amphotericin B	Fluconazole, itraconazole, voriconazole, posaconazole	NA	NA	NA	[50]
(invasive) Oral and systemic candidiasis of HIV patients (n = 1)	Growth on Hicrome *Candida*, germ tube test, clamydospore formation on corn meal agar, and API20C for sugar assimilation	NR	Amphotericin B, fluconazole	NA	NA	NA	NA	[51]
(invasive) Clinical isolate from HIV patient (n = 1)	Culture	microscopy	Fluconazole itraconazole voriconazole amphotericin B	NA	No treatment with antifungal or antimicrobial agents	NA	NA	[52]
(invasive) Oropharyngeal candidiasis in HIV patient (2.9% had C. norvegensis infection) (n = 4)	Culture, germ tube and chlamydosporulation tests	microscopy	ND	ND	Nystatin and clotrimazole	NA	NA	[53]
(invasive) Clinical Isolates from oral cavity, stools/anal, respiratory, urine and, blood/catheter of Candidemia patients (n = 2)	Aux- anogram panel ID 32C Gene sequencing	NR	Itraconazole, voriconazole, amphotericin B, caspofungine, posaconazole	Fluconazole	Fluconazole	Antibiotic	All alive	[54]
(invasive candidiasis) in HCV-related cirrhosis and hepatocarcinoma (n = 1)	blood cultures polymerase chain reaction-sequencing	NR	Anidulafungin	Azoles	Anidulafungin	Vancomycin and piperacillin/tazobactam, and linezolid plus meropenem	Alive	[55]
(invasive candidiasis) in hepatocarcinoma (n = 1)	Blood cultures MALDI-TOF MS	NR	Amphotericin B, itraconazole, voriconazole, caspofungin	Flucytosine Fluconazole	Fluconazole, anidulafungin	Meropenem, vancomycin, amikacin, and prophylactic	Died	[56]
(invasive candidiasis) in peritonitis (n = 1)	Blood culture	CT Scan-abdomen	Voriconazole	NA	Fluconazole, itraconazole	NA	Alive	[57]

MIC: Minimal inhibitory concentrations; NA: Not applicable, because the research is performed on fungal strains/clinical isolates; NR: Not reported; MALDI-TOF MS: matrix-assisted laser desorption ionization mass spectrometry.

**Table 3:** General information and characteristics of candidiasis described for *Candida inconspicua*.

Invasive/Noninvasive Candidiasis (n Human Cases/Strains/Isolates)	Identification Methods	Imaging Test	Antifungal Susceptibility	Antifungal Resistance	Antifungal Treatment	Other Treatments (e.g., Probiotics, Natural Compounds, Antivirals)	Outcome (n)	Reference(s)
(noninvasive) Sample collected from alcoholic beverages (n = NR)	PCR, Sequencing, Enzyme profiling	NR	Amphotericin B and micafungin	Itraconazole, fluonazole,	Itraconazole, fluconazole, amphotericin B and micafungin	No	NA	[40]
(invasive and noninvasive) Samples from oral cavity, anal/stools, urine respiratory, blood/catheter (n = 12)	Ellipsometer test, MIC	NR	Caspofungin	Fluconazole-resistant	Itraconazole, voriconazole, posaconazole isavuconazole, fluconazole, amphotericin B and caspofungin	Broad-spectrum antibiotic	Died (n = 1)	[42]
(invasive) Blood sample (n = 2)	MIC, Fungicidal and fungistatic activity	NR	Echinocandins	Azoles	Fluconazole, caspofungin	No	NA	[43]
(invasive) Systemic mycosis (n = 168)	Antifungal susceptibility and Ellipsometer test, MALDI-TOF MS	NR	Susceptible to echinocandins, polyenes	Azoles	Itraconazole, voriconazole, posaconazole, isavuconazole, fluconazole, Caspofungin, micafungin, anidulafungin, Amphotericin B,	No	NA	[44]

MIC: Minimal inhibitory concentrations; NA: Not applicable, because the research is performed on fungal strains/clinical isolates; NR: Not reported; MALDI-TOF MS: matrix-assisted laser desorption ionization mass spectrometry.

**Table 4:** General information and characteristics of candidiasis described for *C. lypolitica*.

Invasive/Noninvasive Candidiasis (n Human Cases/Strains/Isolates)	Identification Methods	Imaging Test	Antifungal Susceptibility	Antifungal Resistance	Antifungal Treatment	Other Treatments (e.g., Probiotics, Natural Compounds, Antivirals)	Outcome (n)	Reference(s)
(invasive) Clinical isolates (n = 27)	MIC, Fungicidal and fungistatic activity	NR	Amphotericin B, voriconazole	Anidulafungin, micafungin	NR	NA	NR	[50]
(invasive) Catheter-related candidemia/Acute pancreatitis (n = 1)	Blood culture using VITEK 2 YST system	NR	Itraconazole, voriconazole, 5-flucytosine, amphotericin B	NA	Fluconazole, micafungin	Flomoxef	Alive	[67]
(invasive) Catheter-related candidemia/severe oral mucositis (n = 1)	Blood cultures	Microscopy	Fluconazole, itraconazole, amphotericin B and caspofungin	5-flucytosine	Caspofungin, fluconazole	Cefoperazone-sulbactam and amikacin meropenem, teicoplanin	Alive	[70]
(invasive) Bloodstream infections/ (invasive) Clinical isolates (n = 20) (endocarditis, fungemia)	Blood cultures and RNA Sequencing	Microscopy	Amphotericin B, posaconazole, voriconazole, caspofungin	Fluconazole, flucytosine	NR	NA	NR	[71]
(invasive) *Yarrowia lipolytica* fungemia (n = 13)	Blood cultures	Microscopy	Voriconazole, caspofungin, micafungin, anidulafungin, amphotericin B	Fluconazole, itraconazole and posaconazole	Fluconazole	NA	Alive (n = 10) 3 patients died even after treatment with fluconazole	[69]
Invasive candidiasis (n = 16) isolates of *C. lipolytica*	Vitek and API yeast identification systems	NR	Voriconazole echinocandins	Amphotericin B, fluconazole	Amphotericin B	NA	NR	[72]
(invasive) Fungemia (n = 2)	Blood cultures	Microscopy	Voriconazole, caspofungin, amphotericin B, posaconazole, itraconazole, ketoconazole	Fluconazole	Caspofungin, voriconazole	NA	All alive	[73]
(invasive) Catheter-Related Candidemia caused by *C. lipolytica*/blood and the central venous catheter (n = 1)	Blood cultures Biochemical tests	NR	Amphotericin B	Azoles	Trimethoprim-sulfamethoxazole, amphotericin B	Cyclosporine, acyclovir	Died	[74]
(invasive) Catheter-Related Fungemia caused by *C. lipolytica* (n = 3)	Blood culturesBiochemical tests (2) Corneal biopsy culture (1)	NR	Fluconazole, Micafungin (n = 2); Itraconazole, voriconazole, amphotericin B (n = 1)	Fluconazole and 5-flucytosine (n = 1)	Fluconazole	Natamycin, imipenem	All alive	[68]
(invasive) *Candida lipolytica* fungemia (n = 5) (paediatric patients)	Blood cultures	Microscopy	Amphotericin B. Very low susceptibility to fluconazole and itraconazole	NR	Patient 1 and 5: fluconazole Patient 2: no treatment Patient 3 and 4: amphotericin B	NA	All alive	[75]
(invasive) Fungemia/Clinical isolates (n = 58) from blood samples, urine, and vaginal site	Blood culture Biochemical tests DNA sequencing	Microscopy	Fluconazole, posaconazole, itraconazole	Low susceptibility to flucytosine, amphotericin B (n = 3), ketoconazole (n = 2), caspofungin (n = 2), both voriconazole and caspofungin (n = 1) both amphotericin B and ketoconazole (n = 21)	NR	NA	NA	[76]
(invasive) *C. lipolytica* fungemia/septicemia (n = 32)	Blood cultures	Microscopy	Amphotericin B (97% of the isolates), fluconazole (69% of the isolates)	NR	NR	NA	Died (n = 12) Alive (n = 20)	[77]
(invasive) *C. lipolytica* fungemia (n = 1)	Diabetic mellitus, renal failure/blood cultures	Microscopy	Itraconazole, voriconazole, amphotericin B, posaconazole, isavuconazole, anidulafungin	Fluconazole	Caspofungin	NA	Died	[78]
(invasive) *Y. lipolytica* fungemia associated with central venous catheter (n = 14)	Blood cultures sequencing	Microscopy	Caspofungin, micafungin, anidulafungi, amphotericin B	Azoles	Fluconazole	NA	Died (n = 3) Alive (n = 11)	[69]

MIC: Minimal inhibitory concentrations; NA: Not applicable, because the research is performed on fungal strains/clinical isolates; NR: Not reported; MALDI-TOF MS: matrix-assisted laser desorption ionization mass spectrometry.

**Table 5:** General information and characteristics of candidiasis described for *Candida lusitaniae*.

Invasive/Noninvasive Candidiasis (n Human Cases/Strains/Isolates)	Identification Methods	Imaging Test	Antifungal Susceptibility	Antifungal Resistance	Antifungal Treatment	Other Treatments (e.g., Probiotics, Natural Compounds, Antivirals)	Outcome (n)	Reference(s)
(invasive) *C. lusitaniae* (n = 2)	Blood culture Urine culture	Microscopy Chest radiograph	Liposomal Amphotericin-B, Fluconazole, 5-flucytosine	NR	Liposomal Amphotericin B Vancomycin Amphotericin B deoxycholate	NA	Died (n = 1) Alive (n = 1)	[87]
(invasive) *C. lusitaniae* (n = 1)	Blood culture	Microscopy	NR	NR	Fluconazole	Vancomycin, piperacillin/tazobactam, and levofloxacin	Alive	[88]
(invasive) *C. lusitaniae* (n = 8)	Blood culture	Microscopy PCR	Amphotericin B, fluconazole, voriconazole, caspofungin, micafungin, anidulafungin	Fluconazole (Only two isolates)	Amphotericin B Fluconazole, Caspofungin (in 2 only)	Ampicillin and amikacin	Died (n = 3) Alive (n = 5)	[89]
(invasive) *C. lusitaniae* Candidemia (n = 1)	Blood culture	Microscopy	Fluconazole	Amphotericin B	Amphotericin B, fluconazole	NA	Alive	[90]
(invasive) *C. lusitaniae* Candidemia (n = 1)	Blood culture	Microscopy	Amphotericin B, fluconazole 5-flucytosine Itraconazole,	NR	Fluconazole	NA	Alive	[91]
(invasive) *C. lusitaniae* Candidemia (n = 2)	Blood culture Stool culture	Microscopy	AmphotericinB flucytosine	Ketoconazole and miconazole	Amphotericin B ketoconazole	NA	Died (n = 1) Alive (n = 1)	[92]
(invasive) *C. lusitaniae* fingemia (n = 1)	Blood culture Stool culture	Microscopy	NR	AmphotericinB	Fluconazole	Doxycycline clarithromycin	Alive	[93]

MIC: Minimal inhibitory concentrations; NA: Not applicable, because the research is performed on fungal strains/clinical isolates; NR: Not reported; MALDI-TOF MS: matrix-assisted laser desorption ionization mass spectrometry.

**Table 7:** General information and characteristics of the candidiasis described for *Candida rugosa*.

Invasive/Noninvasive Candidiasis (n Human Cases/Strains/Isolates)	Identification Methods	Imaging Test	Antifungal Susceptibility	Antifungal Resistance	Antifungal Treatment	Other Treatments (e.g., Probiotics, Natural Compounds, Antivirals)	Outcome (n)	Reference(s)
(invasive) *C. rugosa* bloodstream infection (n = 19)	Blood culture PCR	Microscopy	5-flucytosine, voriconazole and amphotericin B	Fluconazole (only 4)	Fluconazole amphotericin B	NA	Died (n = 13) Alive (n = 6)	[108]
(invasive) *C. rugosa* bloodstream infection (n = 25)	Blood culture PCR	Microscopy	5-flucytosine, voriconazole	Fluconazole and itraconazole (only 4)	Fluconazole amphotericin B	NA	Died (n = 18) Alive (n = 7)	[109]
(invasive) *C. rugosa* Candidemia (n = 6)	Blood culture PCR	Microscopy	Amphotericin B, fluconazole, and 5-flucytosine	NR	Amphotericin B	NA	Died (n = 5) Alive (n = 1)	[106]
(invasive) *C. rugosa* bloodstream infection (n = 25)	Blood culture	Microscopy	5-flucytosine, voriconazole, fluconazole amphotericin B itraconazole	Voriconazole, flluconazole	Fluconazole amphotericin B	NA	NR	[110]
(invasive) *C. rugosa* fungemia (n = 1)	Blood culture	Microscopy	NR	NR	Fluconazole	NA	Alive	[111]

MIC: Minimal inhibitory concentrations; NA: Not applicable, because the research is performed on fungal strains/clinical isolates; NR: Not reported; MALDI-TOF MS: matrix-assisted laser desorption ionization mass spectrometry.

**Table 8:** General information and characteristics of the candidiasis described for *Candida pelliculosa*.

Invasive/Non- invasive Candidiasis (No. of Human Cases/Strains/Isolates)	Identification Methods	Antifungal Susceptibility	Antifungal Resistance	Antifungal Treatment	Other Treatments (e.g., Probiotics, Natural Compounds, Antivirals)	Outcome (n)	Reference(s)
(invasive) Candidemia (n = 1)	Blood culture, Vitek-2 kit	Fluconazole, flucytosine caspofungin, voriconazole, and amphotericin B	No	Intravenous fluconazole for 2 weeks	NA	Alive (n = 1)	[122]
(noninvasive) Endophthalmitis (n = 1)	Culture of anterior chamber	Intraocular amphotericin B	Topical fluconazole	Multiple intraocular amphotericin B	Netilmicin sulfate cyclopentolate HCl eye drops	Alive (n = 1)	[135]
(noninvasive) Dacryocystitis with cacosmia (n = 1)	fungal hyphae observed on the excised lacrimal sac wall. DNA sequencing	NR	NR	Antifungal agent and washing of the nasolacrimal duct	NA	Alive (n = 1)	[136]
(invasive) Candidemia (n *=* 6)	Blood culture, API-32C and Mini API system, RAPD	2 isolates were resistant to Amphotericin B and low susceptibility to Itraconazole	Fluconazole, voriconazole, and micafungin	Amphotericin B and fluconazole	NA	Alive (n = 5) Died (n = 1)	[125]
(invasive) Candidemia (n = 1)	Blood culture, VITEC2, (ITS) region amplification and sequencing	Amphotericin B, ketoconazole, itraconazole, voriconazole, and fluconazole	No	Fluconazole and amphotericin B	Ceftriaxone, cefepime. and oxacillin	Alive (n = 1)	[137]
(noninvasive) Fungal keratitis (n = 1)	Morphological characteristics and ITS region amplification and sequencing	NR	NR	Topical micafungin	No	Alive (n = 1)	[138]
(invasive) Meningitis in HIV patient caused by *C. pelliculosa* (n = 1)	Phenotypic and molecular methods, Histopathological staining(PAS) from autopsy	NR	NR	NR	Combination antiretroviral therapy (tenofovir, abacavir and atazanavir/ritonavir)	Died (n = 1)	[139]

RAPD: randomly amplified polymorphic DNA; ITS: internal transcribed spacer; NA: not applicable/available; PAS: periodic acid–Schiff.

**Table 9:** General information and characteristics of candidiasis described for *Candida bracarensis* and *Candida intermedia*.

Invasive/Noninvasive Candidiasis (n Human/Cases/Isolate)	Identification Methods	Imaging Test	Antifungal Susceptibility	Antifungal Resistance	Antifungal Treatment	Other Treatments(e.g., Probiotics, Natural Compounds, Antivirals, etc)	Outcome (n)	Reference (s)
***Candida bracarensis***								
(noninvasive) Vulvovaginal candidiasis (n = 1)	API Candida system; ITS1 region and the 5.8S ribosomal RNA gene; sequencing	Microscopy (Germ tube test, chlamydospore test)	Susceptible to nystatin and azoles (fluconazole, itraconazole, miconazole, clotrimazole)	NR	NR	NR	NR	[152]
(invasive) Peripheral neuropathy in type 1 diabetes (patient’s stool positive for *C. bracarensis*) (n = 1)	CHROMagar Candida; multiplex PCR; sequencing; MALDI-TOF MS analysis	NR	Amphotericin B, flucytosine, fluconazole, voriconazole, anidulafungin and caspofungin	Itraconazole (MIC ≥ 32 mg/L), posaconazole (MIC ≥ 32 mg/L)	NR	NR	NR	[161]
***Candida intermedia***								
(invasive) Candidemia (with diabetes bloody sputum, fever, and dyspnea) (n = 1)	API ID32C; molecular identification - D1/D2 domain of the large-subunit 26S rRNA gene	NR	Amphotericin B, flucytosine, fluconazole, itraconazole, miconazole, micafungin	NR	Several antifungals	Antibiotic treatment, mechanical ventilation, steroid therapy	Alive, discharged on the 34th hospital day (n = 1)	[165]

MIC: Minimal Inhibitory Concentrations; NA: Not applicable, because the research is performed on fungal strains/clinical isolates; NR: Not reported; MALDI-TOF MS: matrix-assisted laser desorption ionization mass spectrometry; ITS: internal transcribed spacer.

**Table 11:** General information and characteristics of the candidiasis described for *Candida blankii*.

Invasive/Noninvasive Candidiasis (n Human Cases/Strains/Isolates)	Identification Methods	Imaging Test	Antifungal Susceptibility	Antifungal Resistance	Antifungal Treatment	Other Treatments (e.g., Probiotics, Natural Compounds, Antivirals)	Outcome (n)	Reference(s)
(invasive) *C. blankii* bloodstream infection (n = 1)	Blood sample, CHROMagar Candida, PCR sequencing of rDNA	Microscopy	Voriconazole, itraconazole, posaconazole, amphotericin B, caspofungin, Micafungin, anidulafungin,	Reduced susceptibility to fluconazole b (≥12 μg/mL)	Amphotericin B, fluconazole, caspofungin	Amikacin, ampicillin, cefotaxime, meropenem, teicoplanin, Piperacillin/tazobactam, vancomycin	Died (n = 1)	[192]
(invasive) *C. blankii* fungaemia (n = 9)	ITS and D1/D2 region sequencing	NR	Isavuconazole, posaconazole, itraconazole, voriconazole, micafungin,	Fluconazole had higher MIC (8 μg/mL), Anidulafungin MIC (2 μg/mL) had high MICs	Fluconazole	Broad-spectrum antibiotics	Alive (n = 5), Died (n = 4)	[191]
(invasive) *C. blankii* fungemia and possible endocarditis (n = 1)	Blood culture	NR	Amphotericin B, anidulafungin, 5-flucytosine, itraconazole, micafungin, posaconazole, voriconazole	Fluconazole had higher MIC (16 μg/mL), caspofungin higher MIC (1 μg/mL),	Fluconazole, micafungin and liposomal amphotericin B, voriconazole	Vancomycin, aztreonam, linezolid, daptomycin, meropenem	Alive (n = 1)	[193]
(invasive) *C. blankii* bloodstream infection (n = 1)	Blood cultures, CHROMagar, sequence analysis of the ITS1, and D1D2of the rRNA	NR	Voriconazole, amphotericin B, micafungin	Fluconazole had higher MIC (16 μg/mL), anidulafungin (1 μg/mL)	Liposomal amphotericin B, micafungin	Teicoplanin, meropenem, cotrimoxazole	Alive (n = 1)	[190]

MIC: Minimal inhibitory concentrations; NR: not reported; PCR: polymerase chain reaction; ITS: internal transcribed spacer.

**Table 12:** General information and characteristics of the candidiasis described for *Candida pulcherrima*.

Invasive/Noninvasive Candidiasis (n Human Cases/Strains/Isolates)	Identification Methods	Imaging Test	Antifungal Susceptibility	Antifungal Resistance	Antifungal Treatment	Other treatments (e.g., Probiotics, Natural Compounds, Antivirals)	Outcome (n)	Reference(s)
(invasive) *C.pulcherrima* (n = 1)	Blood culture	Microscopy and ITS sequencing	Amphotericin B lipid complex	NR	Amphotericin B lipid complex	NA	Alive	[195]
(invasive) *C.pulcherimma* (n = 1)	Blood culture	MALDI-TOF MS	Fluconazole	NR	Fluconazole	NA	Alive	[197]
(invasive) *C. pulcherimma* (n = 1)	Blood culture	Microscopy and ITS sequencing	Liposomal amphotericin B, fluconazole, voriconazole, posoconazole, micafungin, anidulafungin	NR	Liposomal amphotericin B and micafungin	NA	Alive	[196]
(invasive) *C. pulcherimma* (n = 1)	Blood cultures	Microscopy and MALDI-TOF MS	Amphotericin B, azoles, echinocandins	NR	Amphotericin B, voriconazole, echinocandins	NA	Alive	[196]

MIC: Minimal inhibitory concentrations; NR: not reported; NA: nonapplicable: PCR: polymerase chain reaction; MALDI-TOF MS: matrix-assisted laser desorption ionization mass spectroscopy; ITS: internal transcribed spacer.

## Data Availability

Not applicable.
